# Evaluation of the Australian first few X household transmission project for COVID-19

**DOI:** 10.1186/s12889-023-14979-3

**Published:** 2023-01-06

**Authors:** Adrian J. Marcato, Miranda Z. Smith, James E. Fielding, Peter D. Massey, Jodie McVernon

**Affiliations:** 1 Department of Infectious Diseases, University of Melbourne, at the Peter Doherty Institute for Infection and Immunity, Victoria, 3000 Australia; 2 Victorian Infectious Diseases Reference Laboratory, Royal Melbourne Hospital, and Department of Infectious Diseases, University of Melbourne, at the Peter Doherty Institute for Infection and Immunity, Victoria, 3000 Australia; 3 College of Medicine and Dentistry, James Cook University, Cairns, QLD Australia; 4 Hunter New England Local Health District, Population Health, Tamworth, NSW Australia

**Keywords:** Pandemic preparedness, Evaluation, Qualitative research, Public health policy, Research in practice, Applied research, Public health, Epidemiology

## Abstract

**Background:**

The Australian First Few X (FFX) Household Transmission Project for COVID-19 was the first prospective, multi-jurisdictional study of its kind in Australia. The project was undertaken as a partnership between federal and state health departments and the Australian Partnership for Preparedness Research on Infectious Disease Emergencies (APPRISE) and was active from April to October 2020.

**Methods:**

We aimed to identify and explore the challenges and strengths of the Australian FFX Project to inform future FFX study development and integration into pandemic preparedness plans. We asked key stakeholders and partners involved with implementation to identify and rank factors relating to the strengths and challenges of project implementation in two rounds of modified Delphi surveys. Key representatives from jurisdictional health departments were then interviewed to contextualise findings within public health processes and information needs to develop a final set of recommendations for FFX study development in Australia.

**Results:**

Four clear recommendations emerged from the evaluation. Future preparedness planning should aim to formalise and embed partnerships between health departments and researchers to help better integrate project data collection into core public health surveillance activities. The development of functional, adaptable protocols with pre-established ethics and governance approvals and investment in national data infrastructure were additional priority areas noted by evaluation participants.

**Conclusion:**

The evaluation provided a great opportunity to consolidate lessons learnt from the Australian FFX Household Transmission Project. The developed recommendations should be incorporated into future pandemic preparedness plans in Australia to enable effective implementation and increase local utility and value of the FFX platform within emergency public health response.

**Supplementary Information:**

The online version contains supplementary material available at 10.1186/s12889-023-14979-3.

## Background

The Australian First Few X (FFX) Household Transmission Project for COVID-19 (henceforth referred to as the FFX Project) was undertaken as a partnership between the Commonwealth Department of Health, state and territory health departments and the Australian Partnership for Preparedness Research on Infectious Disease Emergencies (APPRISE) team based at the University of Melbourne (UoM) and the University of Adelaide (UoA) [1].

The FFX Project was rapidly adapted from the World Health Organization (WHO) Unity Studies FFX and household transmission protocols for COVID-19 [2–4]. In consultation with the national surveillance committee, the WHO protocol was divided into ‘public health’ and ‘research’ elements. This distinction was based on national case and contact management processes in early 2020, at which time neither serology nor genomics were routinely embedded in the public health response or widely available. As such, the FFX project involved epidemiological data and viral swab collection from confirmed cases of COVID-19, and their household contacts, as an enhanced public health surveillance activity. The data collected in the study are detailed further in Additional file 1 and were aligned to the National Notifiable Diseases Surveillance System (NNDSS) fields. The FFX project was expected to help refine and update recommendations for COVID-19 surveillance, characterise the transmission features of SARS-CoV-2 in Australian households, and inform case and contact management models in the early stages of the COVID-19 pandemic.

The New South Wales (NSW), Western Australia (WA), South Australia (SA) and Queensland (QLD) jurisdictional health departments elected to participate in the national FFX project. In Victoria (VIC), the Royal Children’s Hospital (RCH) integrated the FFX project into their broader suite of paediatric hospital-based COVID-19 research. In the absence of a pre-determined and piloted national implementation strategy, unique implementation strategies were developed by the APPRISE team in conjunction with each participating site to account for local staffing capacity and resourcing and data systems. The VIC, Northern Territory (NT), Tasmania (TAS) and Australian Capital Territory (ACT) health departments elected not to participate for various reasons.

Between April and October 2020, 96 households were recruited into the FFX project across the participating centres, with the majority recruited from NSW and the RCH site [1]. While the FFX Project has provided valuable insights into the local epidemiology of COVID-19 particularly regarding the relative transmission of COVID-19 from children, it was challenging to implement a prospective, multi-jurisdictional household transmission study for the first time during a pandemic.

We conducted a formal evaluation of the public health components of the FFX project to identify key challenges and lessons learnt from implementation in 2020. The evaluation outputs will help to inform FFX study development and future pandemic preparedness planning in Australia.

## Methods

The FFX Project evaluation was conducted using a modified Delphi technique in three phases between April and October 2021 [5]. The first round of questions (phase one) was conducted in April–May 2021 via a REDCap online survey. Invitations were sent to individuals (*n* = 11) who were directly involved in the planning, and implementation of the FFX project (these participants are henceforth referred to as implementation partners). There was high turn-over of implementation partners during the FFX project implementation, mainly due to their involvement in pandemic response. To ensure representation across the project group we invited at least one implementation partner from each FFX site to participate in the evaluation.

The phase one questionnaire (Additional file 2) asked a series of questions to identify:

1. The strengths and weaknesses of implementation of the FFX project for COVID-19 in 2020
2. The current and ongoing value of the project in context; and
3. Strategies to improve/enhance future development of the protocol/study in Australia

Responses to each question were compiled and grouped using NVivo 1.4.1 to determine commonly identified themes [6, 7]. Thematic groupings were discussed and agreed by study investigators during the analysis stage.

In the second survey (phase two) conducted in June 2021, implementation partners were asked to rank the themes and ideas identified from the phase one survey and identify the five most important items to them per question. A weighted score was calculated for each question on a continuous scale – items that were ranked most important to an individual scored 5 points, and the least important scored 1 point.

We also engaged the Communicable Disease Network Australia (CDNA) – to nominate representatives from each jurisdictional health department regardless of participation in the project to gain their insights in the phase three interview [8]. All interviews were conducted from late June 2021 – September 2021.

Phase 3 – the grounding phase – was intended to further refine the recommendations from the evaluation. CDNA representatives were provided with a summary of the FFX project to date in late June 2021 and a brief report highlighting the key items raised in the earlier evaluation phases. Representatives were asked similar questions to implementation partners (Additional file 3) and were prompted to reflect and respond from a public health department perspective. Responses from the CDNA representatives were analysed to produce a final set of recommendations for future FFX study development in Australia.

Study data were collected and managed using REDCap electronic data capture tools hosted at the University of Melbourne [9]. Ethics approval for this project was obtained through the University of Melbourne Human Research Ethics Committee (ref: 2021–21,352-16,640–3).

## Results

### Evaluation participants

A total of eleven (100% of invited) implementation partners participated in both phase one and two of the evaluation process. These participants represented each site involved in the implementation of the FFX Project (Table 1). Each Australian state and territory nominated at least one CDNA representative to participate in the phase three interviews – in total there were twelve participants for this evaluation phase. Two implementation partners from the phase one and two surveys participated in the interview alongside their nominated CDNA jurisdictional representative.

**Table 1:** Participants in the evaluation of the FFX project

**Site**^a^	**Implementation partner**	**Number of individuals participating in the phase one and two evaluation survey**	**Number of individuals participating in the phase three interview**
APPRISE^b^	Yes	3	0
Australian Capital Territory	No	0	1
Commonwealth Department of Health	Yes	1	0
New South Wales	Yes	1	2
Northern Territory	No	0	1
Queensland	Yes	0	1
Royal Children’s Hospital, Victoria	Yes	1	0
South Australia	Yes	2	3
Tasmania	No	0	1
Victorian Department of Health	No	0	1
Western Australian Department of Health	Yes	2	2

^a^All sites were embedded within jurisdictional health departments with the exception of the Royal Children’s Hospital and APPRISE

^b^Australian Partnership for Preparedness Research on Infectious Disease Emergencies

### Outcomes from the phase one and two surveys

At least nine distinct items were identified for each question in the phase one survey and one of the questions had twenty-six items. Additional file 4 shows the full list of responses to the phase one survey.

Participants identified nine items relating to the strengths of the FFX Project implementation in the phase two survey. The most highly ranked of these were timely analysis and reporting and the central coordination of the project. Other areas that were deemed important strengths were related to site-specific models of implementation, and solutions to local challenges (Table 2).

**Table 2:** The five highest ranking items per question in the phase two evaluation survey

**Evaluation question**	**Themes identified**	**Weighted score**^a^
***What worked well with the FFX project and why?***	1. Timely analysis and reporting	26
	2. Central coordination by APPRISE	24
	3. Specific models of implementation at each site	23
	4. National funding mechanism and support	21
	5. Collaboration with researchers/research organisations	19
***What didn’t work well with the FFX project and why?***	1. Length and complexity of ethics and governance approval processes	23
	2. Missed opportunity due to delayed start of implementation	22
	3. Missed opportunities due to lack of active engagement from health departments	20
	4. Differing ethical and legislative frameworks across jurisdictions	20
	5. Division into public health and research components led to lost opportunities	17
***What expectations did you have for the FFX project?***	1. The project would deliver on classic FFX objectives and would provide early and rapid epidemiological insights	34
	2. Able to deliver local information on key knowledge gaps	20
	3. There would be more and faster recruitment	18
	4. The data would play a larger role to inform public health advice	16
	A. Relevance of original objectives/research questions waned over time	10
	B. That data would be more accessible and shared in a timely manner	10
***What value do you expect FFX to provide in the ongoing/future FFX study?***	1. Opportunity to conduct further multijurisdictional FFX or related research studies in Australia	25
	2. Further develop surveillance systems and capacity at a national level for pandemic response	21
	3. Developing a process for expedited ethics and governance approvals for future iterations	19
	4. Establishment of a research network within existing public health systems	19
	5. Opportunity to characterise impacts of immunity and vaccination on severity and transmissibility within households	15
***What are the key foundational components/arrangements to have prepared for next time?***	1. Pre-established ethics and governance mechanisms/approvals that cover all study aspects	31
	2. Investments in national data infrastructure to assist with rapid data sharing	16
	3. Clarification of role of FFX studies in national and jurisdictional surveillance plans for pandemic response	15
	4. Maintenance of existing data governance and sharing arrangements from current project	12
	5. Embed within public health work to avoid duplication and participant confusion	10

^a^Items that were ranked and scored from most important (5 points), to least important (1 point)

There were 14 identified challenges raised relating to FFX project implementation. When ranked, the top five items could be grouped into two broad themes relating to challenging and lengthy ethical and governance requirements, and, missed opportunities due to lack of engagement or delayed commencement of the project (Table 2).

Expectations of the FFX Project could be categorised into two main themes after being ranked – it was expected that; 1) the project would provide more information to inform Australia’s pandemic response, and 2) the project would be more useful to inform response in the context of the evolving epidemic (Table 2).

There were 11 themes identified from participants relating to the value of the FFX project. These grouped into two clear themes; using the experience gained from the implementation to inform future FFX studies and providing an opportunity to further investigate COVID-19 through extended analyses including long term serological studies (Table 2).

Twenty-one suggestions for where to target future pandemic preparedness efforts were identified from participants. Once ranked, three themes emerged; pre-establishment of ethics and governance approvals, investment in national data infrastructure to enable the study, and clarifying the role of FFX studies as part of routine public health surveillance activities (Table 2).

Final recommendations for future FFX study implementation:

Four clear recommendations emerged when the outcomes from the phase one and two surveys were discussed with the CDNA representative interviews. The recommendations are further detailed in Additional file 5.

1. Formalise and embed partnerships between involved stakeholders (the Commonwealth Department of Health, jurisdictional public health units and researchers).
2. Integrate FFX data collection into core public health activities and surveillance
3. Develop functional protocols with pre-established funding, ethics, governance, and implementation strategies.
4. Invest in data infrastructure to ensure capacity to rapidly collect, analyse, and report on associated FFX data in a national study.

## Discussion

The FFX project was the first study of its kind in Australia, providing unique insights into the epidemiology of COVID-19 in 2020. Our evaluation explores the key strengths and challenges of implementing the FFX study during a pandemic, including learnings about logistics, ethics, governance and data management. These insights have been developed into four recommendations to inform future iterations of pandemic preparedness plans in Australia [10, 11].

### Partnership between researchers and public health units

The partnership between public health officials and researchers was identified as a key enabler for the implementation of the FFX Project. These partnerships must be further developed and strengthened to ensure well-defined and mature collaborations are available to effectively implement future national FFX studies [12–14]. This process could involve enabling greater opportunities for placements and joint appointments within public health units and research institutes for key implementation partners. Improved understanding of data needs and health systems at a local and national level will allow for rapid development of targeted implementation strategies for FFX studies and other evidence-generating research studies, to help to improve situational awareness in an infectious disease emergency [15].

### Integrate FFX studies into public health response

The FFX project should be recognised as a data platform to generate ‘information for action’, i.e., data that can be used to inform the public health response. The roles and responsibilities of stakeholders such as CDNA, health departments and implementation partners should be clearly defined as part of the planning process to sustainably integrate FFX studies into existing processes.

Building and investing in a dedicated capacity to conduct FFX and related enhanced surveillance investigations is another important aspect to consider [12]. This capacity should facilitate the implementation of FFX studies without impacting on other essential public health work in emergencies. As an example, jurisdictions could explore and determine the feasibility of working with scholars of the Master of Philosophy in Applied Epidemiology, which is the Australian Field Epidemiology Training Program, as part of their required practical placements to provide a dedicated capacity [16]. Such scholars are recognised as having a detailed understanding of the roles and responsibilities of public health units and researchers.

### Develop functional FFX protocols

A pre-established protocol with clear strategies for integration into surveillance and decision-making systems will be necessary for rapid activation in an emergency to allow the study to deliver on its objectives. Protocol development would likely be more appropriate and flexible if ‘modules’ were considered. Modules would enable adaptable implementation in a range of epidemic contexts with varying public health and social measures, testing resources and capacity, funding and personnel to inform understanding of transmission, severity and impact in key populations [17]. An adaptive, modular protocol will increase the capability of FFX studies as an ongoing public health platform to answer evolving information needs as an epidemic progresses.

Health departments would benefit from piloting FFX studies to develop workflows and consider relevant ethics approvals and governance processes in ‘peace-time’ to ensure their utility in an emergency. This will provide a skill building opportunity for the workforce and may have the additional benefit of improving understanding of different diseases (e.g. piloting during an influenza season to understand seasonal dynamics).

Protocol development and piloting must also develop culturally appropriate methods for collecting data in key population groups such as First Nations peoples and those at greater risk of disease (e.g. pregnant women, people in overcrowded housing, culturally and linguistically diverse individuals), to improve equity and health outcomes in outbreaks [18, 19]. Embedding broader governance structures, including representation and leadership from key community and health leaders to guide FFX study adaptation and implementation in addition to pandemic planning is essential to achieve this objective [20–22].

### Invest in national data infrastructure

The collection of high-quality epidemiological data for the FFX study (and other purposes) would be enhanced by the development of a national data system for operational emergency response and research. Australian health care is jointly run by local, state and territory, and federal governments. As a result, there are many differences across jurisdictions particularly in regard to how data are collected, policies regarding data sharing and use, as well as resources and capacity. A national system needs to be flexible to enable the rapid collection of various data types in different epidemiological contexts, but as a minimum, could require the contribution of a core standardised dataset from each participating jurisdiction. Alternatively, future preparedness planning would be further enhanced if there is exploration of how FFX studies and associated data fields can be better embedded within existing infrastructure and data systems at the state and national levels. Consideration of data management aspects for FFX studies in advance of an outbreak will help to improve timeliness of the study by facilitating rapid recruitment and associated data analysis and reporting.

### Limitations

The evaluation took place over a four month period from May–September 2021, approximately a year after the FFX project was actively recruiting. All responses to the phase one and two surveys were received within two weeks of availability. Phase three spanned a three month period from late June to September 2021. Prior to the evaluation period Australia had sustained zero incidence of COVID-19 with periods of localised transmission. From June 2021 (post phase one and two), there were several large outbreaks due to the Delta variant leading to established community transmission in several states.

The perspective of CDNA representatives participating in phase three may have been influenced by the rapidly evolving epidemiology and context. Representatives who participated in interviews when there was no/low community incidence of COVID-19 may have reflected upon the FFX project implementation differently from those who were interviewed once community transmission was established and ongoing. For example, the value of an enhanced epidemiological investigation may have diminished with widespread community transmission as other data provided the required information (i.e. other surveillance data including hospitalisation data, information from overseas, etc.).

### Strengths

Our findings highlight the experience of a large group of stakeholders from varied backgrounds including public health officials at the state and national level, researchers and clinicians. Involvement of members of the core implementation team as well as representatives from each jurisdictional state or territory health department in the evaluation was crucial given the importance of the research and public health partnership needed to facilitate implementation of the FFX project. Our findings supplement a global evaluation of the WHO Unity Studies – the framework from which our study was adapted [23]. Although the evaluation focuses on enhanced surveillance from the WHO/ global perspective, the manuscript discusses similar issues to those identified in our evaluation. We are not aware of any country- or project-specific evaluations of similar household transmission studies for COVID-19.

The modified Delphi process used in the evaluation provided ample opportunity for everyone to reflect and share their opinions of project implementation. The phase one and two surveys were designed to allow people to freely share their ideas, resulting in more accurate understanding of the strengths and weaknesses of the FFX project implementation.

There were some items that were highly ranked in phase two but were raised by few participants in the phase one survey. For example, only one participant stated that the “clear centralised funding mechanism” was a key strength of the study in the phase one survey. This was the most highly ranked item in phase 2 and was deemed important by 64% (7/11) of site implementors. This highlights the strength of our evaluation and the value of multiple survey phases – with robust retention and engagement from participants – to provide a voice to all evaluation participants to identify the collective opinion.

The third phase of interviews added detail about practical solutions to the raised issues from a health department (end-user) perspective, helping to consolidate the findings of the evaluation.

## Conclusion

While some of the site-specific challenges related to our FFX study may not be directly applicable to other countries with unique health systems, there are lessons to be learnt about how general research and enhanced surveillance activities can help to supplement and inform public health pandemic responses.

Platforms like the WHO Unity studies must be continually tested and refined to ensure they are fit-for-purpose in different settings. Overcoming site-specific challenges, such as those described in our evaluation, in peace-time will help enable rapid implementation of FFX studies in response to an infectious disease emergency, and subsequently help to better inform local, national and global public health responses.

This evaluation has provided opportunity for a diverse range of implementation partners to provide rich insights into the strength and limitations of the FFX Project for COVID-19. The findings show that while significant barriers were overcome to implement the project in 2020, more work is needed at both the state and national levels in Australia to better integrate future FFX projects and flexible enhanced surveillance activities within pandemic response plans.

## Supplementary Information

**Additional file 1.**

**Additional file 2.**

**Additional file 3.**

**Additional file 4.**

**Additional file 5.**

## Data Availability

De-identified data supporting the findings of this study are available within the article and supplementary files.

## Abbreviations

FFX: First Few X
APPRISE: Australian Partnership for Preparedness Research on Infectious Disease Emergencies
UoM: University of Melbourne
UoA: University of Adelaide
WHO: World Health Organization
NNDSS: National Notifiable Diseases Surveillance System
NSW: New South Wales
WA: Western Australia
SA: South Australia
QLD: Queensland
VIC: Victoria
RCH: Royal Children’s Hospital
NT: Northern Territory
TAS: Tasmania
ACT: Australian Capital Territory
CDNA: Communicable Disease Network Australia
