# Supplementary material for: Evaluation of the Australian first few X household transmission project for COVID-19
Source: BMC Public Health. 2023 Jan 6;23:41. doi: 10.1186/s12889-023-14979-3 (PMC9817235; doi:10.1186/s12889-023-14979-3)
Supplement: Supplementary file 1 — Additional file 1. [file 12889_2023_14979_MOESM1_ESM.docx]

**Additional File 1: Australian FFX Household Transmission Project Components and details**

| FFX Project component | Summary | Funding source |
| --- | --- | --- |
| Public health component | - Data collection from confirmed cases including status interviews on days 7,14 ± 28 - Data collection from household contacts – daily symptom diaries and status interviews on days 7,14, ± 28 - Specimens at days 0,7,14 ± 28 from household contacts. | Australian Commonwealth Department of Health |
| Research component 1 | - Sequencing of positive swab specimens - Collection and analysis of blood sample after quarantine | APPRISE Centre of Research Excellence |
| Research component 2 – extended follow-up of FFX cohort over 3-year period | - Ongoing serology from current recruits - New FFX recruitment – through FluTracking and Aboriginal Community Controlled Health Organisations pilot study | Australian National Health and Medical Research Council (partnership grant) |
