# Supplementary material for: Evaluation of the Australian first few X household transmission project for COVID-19
Source: BMC Public Health. 2023 Jan 6;23:41. doi: 10.1186/s12889-023-14979-3 (PMC9817235; doi:10.1186/s12889-023-14979-3)
Supplement: Supplementary file 2 — Additional file 2. [file 12889_2023_14979_MOESM2_ESM.docx]

**Additional File 2: Questions asked of implementation partners in the Phase one evaluation survey**

- What worked well with the FFX project and why?
- What didn’t work well with the FFX project and why?
- What expectations did you have for the FFX project (with regards to implementation strategy and outputs) at the beginning of the project and how have these changed over time?
- What value do you expect FFX to provide going forward in the partnership grant or in future FFX iterations?
- What are the key foundational components/arrangements to have prepared for next time? Consider how this would change for another FFX iteration for COVID-19 and for different diseases of pandemic potential (e.g., influenza, Ebola, MERS-CoV)
