# Supplementary material for: Evaluation of the Australian first few X household transmission project for COVID-19
Source: BMC Public Health. 2023 Jan 6;23:41. doi: 10.1186/s12889-023-14979-3 (PMC9817235; doi:10.1186/s12889-023-14979-3)
Supplement: Supplementary file 3 — Additional file 3. [file 12889_2023_14979_MOESM3_ESM.docx]

**Additoional File 3: Questions asked of Communicable Disease Network Australia representatives in the Phase three evaluation interview**

- How aware were you of the outputs of the FFX project?
- How did you access and use the outputs of FFX project to inform the public health response?
- What expectations did you have for the FFX project (with regards to implementation strategy and outputs) at the beginning of the project and how have these changed over time?
- What value do you expect FFX to provide going forward in the partnership grant or in future FFX iterations?
- What are the key foundational components/arrangements to have prepared for next time? Consider how this would change for another FFX iteration for COVID-19 and for different diseases of pandemic potential (e.g., influenza, Ebola, MERS-CoV)
