# Supplementary material for: Evaluation of the Australian first few X household transmission project for COVID-19
Source: BMC Public Health. 2023 Jan 6;23:41. doi: 10.1186/s12889-023-14979-3 (PMC9817235; doi:10.1186/s12889-023-14979-3)
Supplement: Supplementary file 4 — Additional file 4. [file 12889_2023_14979_MOESM4_ESM.docx]

**Additional File 4: Themes identified from the Phase 1 Evaluation survey with FFX project implementation partners**

1. What worked well with the FFX project and why?

- Collaboration with researchers/research organisations to enable implementation
- Central coordination by APPRISE (including organisation and conduct of regular project meetings with sites, provision of support through implementation, communication, and availability)
- Existing relationships between the Office of Health Protection (Commonwealth) and APPRISE/UoM research team
- Rapid adaptation of the WHO FFX protocol to be used
- Data governance arrangements
- National funding mechanism and support
- Jurisdiction specific models of implementation to address unique challenges at each site
- National data repository and REDCap collection platform
- Timely analysis and reporting providing unique insights into household transmission in Australia

1. What didn’t work well with the FFX project and why?

- Differing ethical and legislative frameworks across jurisdictions for the collection and analysis of case and contact data
- Length and complexity of ethics and site-specific governance approval processes which impacted on ability to undertake research components
- Issues in set-up relating to swab accessibility, engagement with pathology providers or other jurisdiction specific issues
- Eligibility criteria too stringent, i.e., excluded if household spoke a language other than English
- Lack of national data collection system – bespoke solutions per site which led to some duplication of work for entry into national repository
- Storage of data and analysis within the virtual machine proved challenging
- Missed opportunity due to delayed start of implementation or delay in pivoting project objectives/methods
- Limited outputs and data – due to lack of eligible cases and differences in site participation/engagement
- Further information needed to be collected to reflect epidemiology of COVID-19 e.g., additional household exposures/measures and how they varied over time
- Limited capacity and resourcing in health departments
- Missed opportunities due to lack of active engagement from health departments or representatives
- Division into public health and research components led to lost opportunities and created some tensions
- Concerns for participants (e.g., confusion about the distinction between mandatory public health and research components)
- Lack of preparedness – untested systems and processes were hard to implement in multi-jurisdictional study for the first time in a pandemic

1. What expectations did you have for the FFX project (with regards to implementation strategy and outputs) at the beginning of the project and how have these changed over time?

- The FFX project would deliver on classic FFX objectives and would provide early and rapid epidemiological insights, i.e., characterisation of clinical course of disease, local epidemiology
- Able to deliver local information on key knowledge gaps i.e., the relative contributions of adult and children to transmission within households
- Greater collaboration from all sites
- Became apparent that we wouldn’t have enough data to provide certainty on our estimates of key parameters
- The data would play a larger role to inform public health advice
- Relevance of original objectives/research questions waned over time - wasn’t useful given no large-scale community transmission and as information came in from other sources
- Became apparent over time that most outputs will be long-term and more academic
- There would be more and faster recruitment
- Shifting to a case study of FFX project implementation – learning how we can implement these studies
- The massive limitations /recruitment bias
- Realities of research recruitment
- Unable to contribute to research outputs
- More support in completing ethics and governance processes
- That data would be more accessible and shared in a more timely and collaborative manner
- To finalise an ethically approved protocol across participating sites
- Expectation of further development of protocols and processes before further established community transmission of COVID-19 to contribute to local/international knowledge and inform ongoing public health and immunisation strategies.
- Unsure of expectations given this was the first time implementing an FFX in Australia across multiple jurisdictions

1. What value do you expect FFX to provide going forward in the partnership grant or in future FFX iterations?

- Additional analyses incorporating genomics and serology
- Opportunity to characterise impacts of prior immunity and vaccination on severity and transmissibility of emerging variants within household units in longitudinal study
- Understanding how the FFX platform can be adapted for use in First Nations communities
- Establishment of a research network within existing public health systems
- Further strengthen relationships between public health representatives and researchers
- Opportunity to conduct further multijurisdictional FFX or other related research studies in Australia
- Consolidating a process for the management and implementation of FFX studies
- Further develop surveillance systems and capacity at a national level to help understand the epidemiology of pathogens
- Clarity around jurisdiction specific legislation for the collection of data and biological specimens on cases and their contacts
- Developing a process to expedite ethics and governance approvals for future iterations
- Not much added value

1. What are the key foundational components/arrangements to have prepared for next time? Consider how this would change for another FFX iteration for COVID-19 and for different diseases of pandemic potential (e.g., influenza, Ebola, MERS-CoV)

- Investments in national data infrastructure to assist with rapid data sharing
- Development of a unified data collection platform – separate system for emergency public health research to ease collection/transfer requirements
- Harmonisation of data fields
- Pre-specified communication channels and focal points
- Consider what activities and capabilities might be required for different types of pathogens (e.g., airborne, droplet, fomite, vector)
- Maintenance of existing data governance and sharing arrangements from current project
- Investment into data analytics capacity (including testing and development of code)
- Pre-established ethics and governance mechanisms/approvals that are agreed to by health departments and cover all study aspects under one pathway (via public health act or full ethics approval)
- Identification of potential research partners to support all jurisdictions and further development of this workforce
- Developed protocols and other FFX study documentation
- Inclusion of biobanking with a broad remit to cover all eventualities for an emerging pathogen
- Inclusion of additional data fields; screening log to understand selection biases, non-recruited households
- Aim to embed within public health work to avoid duplication and participant confusion, encourage timely identification of case
- Developed capacity and resourcing (at a national level) to be provided to states
- Clear buy-in from all implementation partners to help further develop these studies and to ensure importance and role of FFX is clear as a public health activity
- Further piloting and refinement of FFX protocols before the next pandemic (regular exercising)
- Pre-engagement of national bodies (CDNA/PHLN) to facilitate cross-jurisdictional information sharing and agreeing to study components for any given pathogen in context in real time and within capacity of existing systems
- Implementation plan including agreed options for funding structure and clear responsibilities for all involved
- Exploration of the way Australian investigators can contribute to design and implementation of similar studies in our region and part of our capacity building commitment
- Pre-agreed information sharing with WHO to enable our well-resourced country to contribute to global knowledge gain in real time
- Clarification of role of FFX studies in broader surveillance plans (national and jurisdictional level) for pandemic response
- Understanding the nature of a threat (pathogen) and public health response that would suggest an FFX type project would be able to provide rapid and high-quality pandemic intelligence
- Exploration of the way Australian investigators can contribute to design and implementation of similar studies in our region and part of our capacity building commitment
- Pre-agreed information sharing with WHO to enable our well-resourced country to contribute to global knowledge gain in real time
