# Supplementary material for: Evaluation of the Australian first few X household transmission project for COVID-19
Source: BMC Public Health. 2023 Jan 6;23:41. doi: 10.1186/s12889-023-14979-3 (PMC9817235; doi:10.1186/s12889-023-14979-3)
Supplement: Supplementary file 5 — Additional file 5. [file 12889_2023_14979_MOESM5_ESM.docx]

**Additional File 5: Details of evaluation recommendations**

Formalise and embed partnerships between involved stakeholders (the Commonwealth Department of Health, jurisdictional public health units and researchers).

The partnership between public health officials and researchers was pivotal to the implementation of the Australian FFX Household Transmission Project for COVID-19. These partnerships should be further developed and strengthened to ensure well-defined and mature collaborations are available to effectively implement future FFX studies that can help inform the public health response in a timely manner.

Further considerations for implementing this recommendation include:

- Integrating opportunities and placements within jurisdictional and national public health units for researchers and public health staff. This could include opportunities for collaboration between state and federal health departments, opportunities for researcher placements within public health units as well as research collaboration opportunities for public health staff.
- Joint appointments (as per clinicians) could be considered to improve trust and knowledge exchange between researchers and public health staff from different jurisdictions to:
- Improve understanding of public health needs and research requirements in a pandemic or epidemic.
- Improve understanding of specific health systems and their associated strengths and challenges.
- Clarifying and defining the role of researchers in pandemic response (in general). Projects that generate information for action should preferably involve a health lead and be performed through health processes and governance mechanisms. Involvement of external organisations (such as the University of Melbourne) can be uncomfortable for some jurisdictions and can change perception of projects from operational public health response to research, even where there is clear endorsement of research collaboration to enhance public health response.

Integrate FFX data collection into core public health activities and surveillance

For FFX studies to inform the public health response in a timely manner, further clarification of their role as a public health and research partnership is required. Protocol development should consider how FFX studies can be implemented in different epidemic contexts to answer current, emerging, and ongoing information needs. The roles and responsibilities of stakeholders and implementors should be clearly defined as part of the planning process.

Specific considerations to achieve this recommendation include:

- Clarifying and communicating the purpose of FFX studies at the national and jurisdictional level to:
- Increase understanding that FFX activities generate ‘information for action’, i.e., the data that is collected can be used to inform the public health response.
- Consider the utility of the FFX platform for individual jurisdictions.
- Explore whether existing infrastructure and data can be used to address information needs through better sharing and use of existing platforms.
- Enabling the FFX platform to adapt to changes in the local epidemiological situation, altered resource availability and emerging evidence.
- Clarifying the role of FFX as a public health and/or research activity in different pandemic phases.
- Considering the development of a ‘modular’ FFX platform with multiple sub-studies to answer specific question packages depending on information needs. For example:
- Transmission studies – investigate transmission dynamics and severity in contacts in closed settings such as households, workplaces, hospitals.
- Long-term follow-up – characterise long term clinical effects of infection.
- Serological studies – analyse the effect of (re)exposure, vaccination etc. on the development of immunity.
- Studies in unique cohorts or demographics – determine if transmission and clinical severity is different in specific cohorts.
- Exploring how research can be sustainably integrated into public health units to enable FFX data collection and follow-up:
- Ensuring that public health work can occur without hindering additional but aligned research such as FFX conduct. Some jurisdictions suggested considering whether aspects of FFX could be conducted retrospectively, and proposed a modified process:
  - Case interviews and isolation of household contacts occurring as a public health activity.
  - Researchers assisting public health units with the ongoing management of household contacts in line with public health advice including symptom checks, specimen collection and testing, and reporting of results. Participants could be consented into the study at this point.
- Building a dedicated capacity to conduct FFX and other enhanced surveillance investigations. For example, jurisdictions are familiar and comfortable with Master of Philosophy in Applied Epidemiology (MAE) students. This is a well trusted and recognised pathway.

Develop functional protocols with pre-established funding, ethics, governance, and implementation strategies.

A pre-established protocol with clear strategies for integration into surveillance and decision-making systems will be necessary for rapid deployment. This will also help to meet expectations for protocol activation to provide early situational information to inform the public health response. Many jurisdictions were overwhelmed with the public health response to COVID-19 in early 2020. The FFX project was therefore not feasible within public health or laboratory capacity at the time due to a lack of these pre-arrangements.

Specific considerations to achieve this recommendation include:

- Consolidating processes for study governance within existing public health structures.
- Establishing secure and pre-agreed funding arrangements.
- Seeking ethics and site-specific governance pre-approvals.
- Continually refining and testing the protocol in peacetime:
- Considering how the study can be implemented in a range of scenarios accounting for differences in disease dynamics, capacity and public health and social measures
- Iteratively implementing the study across each jurisdiction to determine how processes work within different surveillance systems.
- Utilising the platform to update knowledge for different diseases (i.e., influenza behaviour in different seasons).
- Offering skill building opportunities for those involved.
- Exploring barriers to implementation and developing working solutions for diverse communities and settings, including:
- Embedding broader governance structures, including representation and leadership from key community and health leaders to guide FFX study adaptation, planning and implementation.
- Partnering with First Nations researchers and organisations to explore how an FFX study could be undertaken in a First Nations context.
- Partnering with community groups who may be disproportionately or differently affected by disease (e.g. those in specific occupations, people in overcrowded housing, recent migrants and other culturally and linguistically diverse communities).

Invest in data infrastructure to ensure capacity to rapidly collect, analyse, and report on associated FFX data in a national study.

The collection of high-quality epidemiological data for the FFX study would be facilitated by the development of a national data system for operational emergency response. The required data needs to be collected in a harmonised way before being uploaded into a relevant national database. This will improve the timeliness of data analysis and reporting and ensure greater benefit of project outputs in informing the public health response.

Specific considerations to achieve this recommendation include:

- Developing a harmonised national data system that is piloted, trialled, and understood by all jurisdictions:
- Jurisdictions should collect their data in the required format for aggregation into the national dataset.
- The required data should be part of standard public health surveillance, i.e., the minimum data required for the FFX analysis should be collected to enable contribution.
- The national system (as well as state systems) should be flexible to enable the collection of different data in response to a changing epidemiological situation.
- Strengthening chains of communication to increase awareness of study outcomes:
- At the national level this could be achieved through public health networks.
- At the jurisdictional level this would need to be tailored to account for different internal structures and complexities.
- Reviewing FFX reporting processes to improve accessibility and utility of study results:
- Considering rapid analysis of this data to inform the public health response in real-time.
- Ensuring multiple methods for reporting to diverse audiences (i.e., Commonwealth, CDNA and jurisdictional representatives, contact tracing teams, medical teams, participants, and the public).
